# Intrinsic brain functional connectivity mediates the relationship between psychological resilience and cognitive decline in ageing

**DOI:** 10.1007/s11357-025-01529-5

**Published:** 2025-02-03

**Authors:** Menglu Chen, Mengxia Gao, Junji Ma, Tatia M. C. Lee

**Affiliations:** 1 State Key Laboratory of Brain and Cognitive Sciences, The University of Hong Kong, Hong Kong SAR, China; 2 Laboratory of Neuropsychology & Human Neuroscience, The University of Hong Kong, Hong Kong SAR, China

**Keywords:** Psychological resilience, Functional connectivity, Processing speed, Resting-state fMRI, Ageing

## Abstract

Ageing individuals often experience cognitive decline and intrinsic functional connectivity (FC) changes. Psychological resilience, a personality trait that reflects the capacity to adapt and cope with age-related challenges, plays a key role in mitigating cognitive decline. In this study involving 101 older adults, we investigated how psychological resilience influences cognitive decline measured by processing speed. Particularly, we obtained resting-state functional magnetic resonance imaging (fMRI) to assess how intrinsic FC, represented by degree centrality, modulates the relationship between resilience and processing speed. Our results indicated while psychological resilience positively predicted processing speed, this relationship was mainly driven by education. Additionally, the degree centrality of both thalamus and caudate negatively correlated with processing speed and resilience. Notably, the degree centrality of both thalamus and caudate significantly mediated the relationship between resilience and processing speed. These findings suggest that psychological resilience could protect against age-related cognitive decline via its influence on FC in the thalamus and caudate, highlighting these areas as potential intervention targets for reducing cognitive decline in ageing people.

**Supplementary Information:**

The online version contains supplementary material available at 10.1007/s11357-025-01529-5.

## Introduction

Ageing is often accompanied by changes in intrinsic functional connectivity (FC) of the brain and cognitive decline in human beings. Specifically, FC progressively diminishes with increasing age in older adults [10, 45], leading to cognitive disruptions, including information processing speed, attention, and memory [2]. These changes present substantial challenges and increase the burden on our rapidly ageing society [7, 8]. Interestingly, individuals with high psychological resilience seem to experience slower cognitive decline later in life. This intriguing observation suggests the possibility of maintaining or perhaps even enhancing cognitive functioning in older individuals through the modulation of psychological resilience [39, 59, 72]. Moreover, it is crucial to identify the key brain areas and the FC that impact resilience and cognitive decline in older people. Understanding these mechanisms not only clarifies the brain dynamics underlying resilience but also highlights specific regions that could be targeted in interventions aimed at reducing cognitive decline. Furthermore, these targeted interventions could potentially enhance resilience and protect ageing individuals from cognitive decline. One of the cognitive domains most affected by ageing and relevant to resilience is processing speed [4]. This fundamental aspect of cognitive ability serves as a prominent biomarker for cognitive decline in older adults [47, 66]. In this study, we aim to examine how psychological resilience affects cognitive decline, focusing on processing speed, and investigate the role of FC in this relationship. Given the graph theory approach in functional magnetic resonance imaging (fMRI) is widely applied to evaluate FC patterns in ageing populations, it is an ideal method for our analysis [2, 41, 54, 56].

Psychological resilience is generally described as the capacity to effectively recover from challenges, stressors, substantial risks and significant threats, or traumatic experiences [13, 23, 76]. It has also been characterized as a stable personal trait manifesting even in the absence of a stressful situation [27, 48]. Previous studies have demonstrated that resilience could predict overall cognitive performance with higher resilience correlates with better cognitive functions in both adolescents and older adults [58, 67, 72]. Specifically, processing speed is an important cognitive function, declining dramatically with age [24, 60]. It also emerges as a fundamental mediator of age-related differences in a variety of cognitive functions and is essential for reflecting neurological pathologies in older adults [1, 11, 26]. Since resilience may serve as a protective trait for cognitive decline and processing speed is foundation to age-related cognitive functions, an important question remains whether resilience plays a protective role in the processing speed of older individuals. To address this question, studying the relationship between resilience and processing speed in older adults can enhance our understanding of the proactive factors influencing cognitive decline. Processing speed can be assessed using various tests that involve simple or complex scenarios [9]. In this study, we used the Symbol Digit Modalities Test (SDMT), which focuses on simple scenarios, to measure processing speed in older adults. The SDMT was selected for its strong validity, ease of administration, and widespread application among ageing populations [44, 52, 68].

Existing neuroimaging studies have independently investigated the neural substrates underpinning resilience and processing speed, revealing significant insights. For resilience, a task-based fMRI study with 200 nonpatient young adults indicated that the interactions between recent life stress and activation in the striatum predict resilience-related positive affect. Resilience was represented by positive affect in response to recent life stress. This highlights the involvement of reward-related brain regions in resilience [20]. Neurobiological evidence points to critical brain systems involved in stress response and reward experience, implicating regions such as the ventral medial prefrontal cortex, amygdala, thalamus, hippocampus, anterior cingulate cortex, insula, and striatum [14, 19]. Notably, evidence from two case studies using T1 MRI indicated that the caudate, a subregion of the striatum, plays a key role in the corticostriatal circuit, connecting cognitive and emotional functions with other cortical regions [73]. Research using T1-weighted MRI on 80 participants (40 with Parkinson’s disease and 40 age-matched controls) has demonstrated that the caudate’s volume is associated with mental flexibility and processing speed. In this study, the processing speed was measured by the Digit Symbol and Symbol Search subtests from the Wechsler Adult Intelligence Scale-Third Edition [28]. Both a task-based fMRI study with 40 participants and a resting-state fMRI study with 99 older adults further suggested that processing speed, measured by the Arrow Task and the SDMT, involves multiple brain regions, including the dorsal lateral prefrontal cortex, medial frontal cortex, thalamus, hippocampus, insula, caudate, and sensorimotor system [11, 26]. A study combining the T1-weighted structure MRI and resting-state fMRI with 181 older adults suggested that the interaction between the caudate and hippocampus is also associated with episodic memory, supporting cognitive functions in older adults [74]. Given these findings, the thalamus, hippocampus, caudate, and insula emerge as critical regions for both resilience and processing speed, making them ideal targets for investigating the neural mechanisms linking resilience and processing speed. To explore this relationship, we applied nodal degree centrality, a graph theory measure reflecting the number and strength of direct functional connectivity between a brain region and other regions, thereby representing its network integration role [75, 76]. Degree centrality was applied because it provided a straightforward and interpretable measure of a node’s connectivity and it is a widely validated measure in neuroscience, offering clear neurobiological interpretations. By examining the degree centrality of the thalamus, hippocampus, caudate, and insula, we aim to uncover the intrinsic functional connectivity roles of these brain regions [77, 75] –––– and the neural mechanisms behind the relationship between resilience and processing speed.

To address the aforementioned research gaps, we proposed the following hypotheses: (1) resilience would be a protective factor in the cognitive function of ageing individuals [59, 82], specifically by affecting the performance of processing speed in older adults, and (2) the effect of resilience on processing speed may be mediated by the intrinsic degree centrality in the thalamus, hippocampus, caudate, and insula. To test these hypotheses, we collected resting-state fMRI data from healthy older adults and assessed their resilience and processing speed outside of the scanner. Resilience was assessed using the Chinese version of the Dispositional Psychological Resilience Scale (DRS-15), a well-validated and reliable measure that captures the key aspects of psychological resilience, including control, commitment, and challenge [3, 76, 81]. We then extracted the degree centrality of the thalamus, hippocampus, caudate, and insula based on graph theory. Subsequently, we conducted partial Spearman correlation analyses to explore the associations between resilience and processing speed, including age, sex, and education as covariates. Finally, we implemented mediation analyses to test whether the intrinsic degree centrality of these brain regions would play a mediating role in the relationship between resilience and processing speed in healthy older adults.

## Materials and methods

### Participants

Participants were recruited from the local community in Hong Kong with the help of various nongovernmental organizations and via advertisements posted in public places. This study included 101 right-handed, healthy older adults (73 females and 28 males), aged between 60 to 79 years (mean age = 66.7 years, SD = 4.6 years). The inclusion criteria for participants were as follows: (1) not cognitively impaired, defined by a Montreal Cognitive Assessment (MoCA)—Hong Kong version score of 22 or above, based on the cutoff for older Chinese adults in Hong Kong [16]; (2) no depression, as defined by a Geriatric Depression Scale (15-item) score of 7 or below [5]; (3) no reported history of present or past neurological (e.g., brain injuries, stroke, epilepsy) or psychiatric (e.g. depression, anxiety, schizophrenia) conditions; and (4) completion of resting-state scanning with minimal head motion, defined as absolute head motion ≤ 2 mm translation and ≤ 2° rotation, or a mean frame-wise displacement (FD) ≤ 0.2 mm [73, 74]. Written informed consent was obtained from all participants. Ethical approval for this study was granted by the Human Research Ethics Committee of the University of Hong Kong. We conducted additional analyses to explore potential effects of sex differences (see Supplementary information (SI) Table.S4).

### Psychological resilience assessment

The Chinese version of the Dispositional Psychological Resilience Scale (DRS-15) was applied to assess participants’ psychological resilience. It consists of 15 items, each scored on a four-point scale ranging from 0 (not at all true) to 3 (completely true). The items comprise both positive and negative descriptions, such as “I enjoy the challenge when I have to do more than one thing at a time” or “Life in general is boring for me.” The overall score ranges from 0 to 45, with higher scores indicating a greater level of resilience. The Chinese version of the scale has demonstrated reliability and validity in Hong Kong, with a Cronbach’s *α* coefficient of 0.78 [64].

### Processing speed assessment

Processing speed was evaluated by the Chinese version of the Symbol Digit Modalities Test (SDMT) [78, 79]. During this test, participants were required to match a series of nine geometric designs with the corresponding numbers 1 to 9 according to the key presented. Participants were required to substitute the symbols with their respective numbers in sequence by reading aloud the number as quickly as they could within 90 s. The total number of accurately matched items was recorded as the SDMT score, which served as an indicator of the participants’ processing speed ability.

### Imaging data acquisition

Whole-brain images were acquired from a Philips 3.0 T MRI scanner at the University of Hong Kong. The resting-state fMRI scans consisting of 240 volumes were acquired using a single-shot gradient-echo multislice echo-planar imaging (EPI) pulse sequence (slice number = 32; slice thickness = 4 mm without inter-slice gap; TR = 2000 ms; TE = 30 ms; flip angle = 90°; voxel size = 1.6 × 1.6 × 4 mm^3^, FOV = 230 × 230 mm^2^) in about 8 min. In addition, each participant’s high-resolution anatomical images were acquired through three-dimensional sagittal T1-weighted magnetization-prepared rapid gradient echo (MPARGE) with a total of 137 slices (slice thickness = 1.2 mm, TR = 6.64 ms, TE = 3.1 ms, flip angle = 9°, voxel size = 1.2 × 1.0 × 1.0 mm^3^, FOV = 256 × 256 mm^2^).

### Imaging preprocessing

All the images were preprocessed using statistical parametric mapping (SPM12, https://www.fil.ion.ucl.ac.uk/spm/software/spm12/) [78] based on MATLAB software platform (MATLAB R2022a; MathWorks Inc., Natick, MA, USA) and DPABI 5.3 [80]. For the resting-state fMRI data, the first five volumes of resting-state scans were discarded for signal equilibrium and participants’ adaptation to scanner noise. The remaining images were then corrected for slice acquisition timing and realigned for head motion. Nuisance regressors, including mean signals from white matter, cerebral-spinal fluid signals, and global signals, as well as the Friston 24-motion parameters (six motion parameters, six motion derivatives, and their squares), were regressed out from the data. Additionally, head motion scrubbing was performed: volume with a mean frame-wise displacement (FD) > 0.5 mm was added as a covariate, and the one volume prior to this volume and the two volumes after this volume were also added as covariates [77]. Participants were excluded with absolute head motion ≤ 2 mm translation and ≤ 2° rotation, or a mean frame-wise displacement (FD) ≤ 0.2 mm [73, 74]. Finally, images were spatially smoothed using an isotropic 3D Gaussian kernel of 6-mm full-width-at-half-maximum (FWHM) and temporally smoothed using the frequency bandwidth of 0.01–0.1 Hz.

### Functional network construction and regions of interest

Based on the literature on processing speed and resilience, we chose the brain regions which were found to be associated with both processing speed and resilience as our regions of interest (ROI). They were namely the thalamus, hippocampus, caudate, and insula. Brain functional network was constructed with nodes defined using the automated anatomical (AAL-90) template which included the four regions of interest (ROIs) that we identified from the literature [35]. For each participant, we extracted the mean time series of each node in the AAL-90 template by averaging the time series of all voxels in each node from their resting-state imaging data. We then correlated the mean time series of each pair of nodes using Pearson correlation, a widely used method in neuroimaging that quantifies the linear relationship between brain regions, providing a clear and interpretable measure of connectivity [50, 53]. Subsequently, we applied Fisher’s r-to-z transformation to the correlation coefficients to construct one 90 × 90 network matrix for each participant. We conducted supplementary analyses to explore correlations between degree centrality and processing speed across all AAL brain regions, examining potential processing speed-related areas (see SI).

### Calculation of intrinsic degree centrality

As one of the most frequently used metrics in network analysis, degree centrality represents the overall connectivity of a given node to all other nodes in a brain network, reflecting its influence and importance in the functional network. The degree centrality is calculated by the sum of all edges connected to the node [6, 49]. A higher degree centrality indicates that a node has more and/or higher strength connections with other nodes, suggesting its pivotal role in information communication. To calculate the degree centrality of the thalamus, hippocampus, caudate, and insula, we entered the 90 × 90 network matrix of each participant into GRETNA for network analysis (https://www.nitrc.org/projects/gretna/). We employed a weighted network approach which reflects the strengths of the connectivity between brain nodes, considering only the positive values of the correlations between nodes. Specifically, we applied a range of sparsity thresholds from 0.05 to 0.5 in increments of 0.05, averaging the results across these 10 thresholds to ensure a robust and comprehensive assessment of network properties [25, 43]. We combined the degree centrality values extracted from both the left and right hemispheres into a single value by averaging their degree centrality. The degree centrality of node i (*N*_i_) was defined as follows:

$$dc\left({N}_{i}\right){\sum }_{j=1}^{n}{x}_{\text{ij}}\left(i\ne j\right)$$

where *n* is the total number of the nodes in network and ${\sum }_{j=1}^{n}{x}_{\text{ij}}$ is the sum of the connections directly connected with node *i*. The variable *j* represented all other brain region nodes in the network, excluding node *i*.

We also calculated additional graph theory metrics, including nodal efficiency and betweenness centrality analyses (see SI, Table S2, S3).

### Statistical analysis and mediation analysis

First, we conducted a partial Spearman correlation to investigate the relationship between the degree centrality of the four brain regions, processing speed, and resilience, controlling for potential confounding factors such as age, sex, and education. We applied false discovery rate (FDR) correction with a significance level of *p* < 0.05 to account for multiple comparisons across the four regions. Significant brain nodes identified through these analyses were then subjected to further investigation. Next, we examined whether the resting-state degree centrality of the four regions of interest (ROIs) mediated the relationship between resilience and processing speed. We conducted mediation model analyses using SPSS version 21 with the PROCESS macro (IBM Corp., Armonk, NY) [81]. The significance of the indirect (mediated) effect was assessed using 5000 bias-corrected bootstrapping [82], and the indirect effect was considered significant if the 95% confidence interval (CI) did not include zero. In these mediation analyses, resilience was treated as the independent variable (*X*), the degree centrality of ROIs as the mediator (*M*), and processing speed as the dependent variable (*Y*). Additionally, we included age, sex, and education as covariates to account for their potential effects on the relationships being studied.

## –Results

### Demographics and processing speed in older adults

A total of 101 older adults (see Table 1 for more characteristics) were included in the final analyses in this study (Fig.S1A and B). We use the scores of oral SDMT as the indicator of participants’ processing speed and their scores range from 25 to 82 (mean score ± SD = 53.9 ± 10.7). Furthermore, older adults’ hardiness scores were used as an index of psychological resilience, ranging from 11 to 44 (mean ± SD = 28.4 ± 5.63).

**Table 1:** Participants’ demographic

	Participants
Total number	101
Age in years (mean ± SD)	66.7 ± 4.6
Age range	60–79
Sex	73 F/28 M
Education years (mean ± SD)	12.2 ± 4.67
Processing speed (SDMT score) (mean ± SD)	53.9 ± 10.7
Resilience score (mean ± SD)	28.4 ± 5.63

*M* male, *F* female, *SD* standard deviation

To examine the processing speed of older adults, we conducted a Spearman correlation analysis between processing speed and the demographic including age, sex, and years of education. We observed that the processing speed had an inverse correlation with age and education years, as it negatively correlated with age (*rho* =  − 0.55, *p* < 0.001), but positively correlated with education years (*rho* = 0.35, *p* < 0.001), and no correlation with sex (*rho* =  − 0.07, *p* = 0.464).

A partial Spearman correlation analysis was further conducted to investigate the association between psychological resilience and processing speed, including age, sex, and/or education as covariates. A significant positive correlation result was observed between resilience and processing speed in older adults (*rho* = 0.21, *p* = 0.04) while controlling for individuals’ age and sex. However, when we added education years as another covariate in the partial Spearman correlation, no significant correlation was revealed (*rho* = 0.13, *p* = 0.219). It is worth noting that resilience showed a positive correlation with education years (*rho* = 0.38, *p* < 0.001), and this is consistent with previous findings [58] (Table 2). These findings suggest that processing speed in this healthy ageing population is affected by their psychological resilience when not controlling for the effect of education.

**Table 2:** Partial Spearman correlation results between resilience and processing speed

Partial Spearman correlation	*rho*	*p*_value	Covariates
Resilience and processing speed	0.21	0.04	Age and sex
Resilience and processing speed	0.13	0.219	Age, sex, and education
Resilience and education	0.38	< 0.001	Age and sex

Partial Spearman correlation results revealed that resilience had no direct association with processing speed when controlling for education years, while resilience showed a correlation with education years in healthy older adults.

### Association between intrinsic thalamus and caudate degree centrality and processing speed

**Fig. 1:**
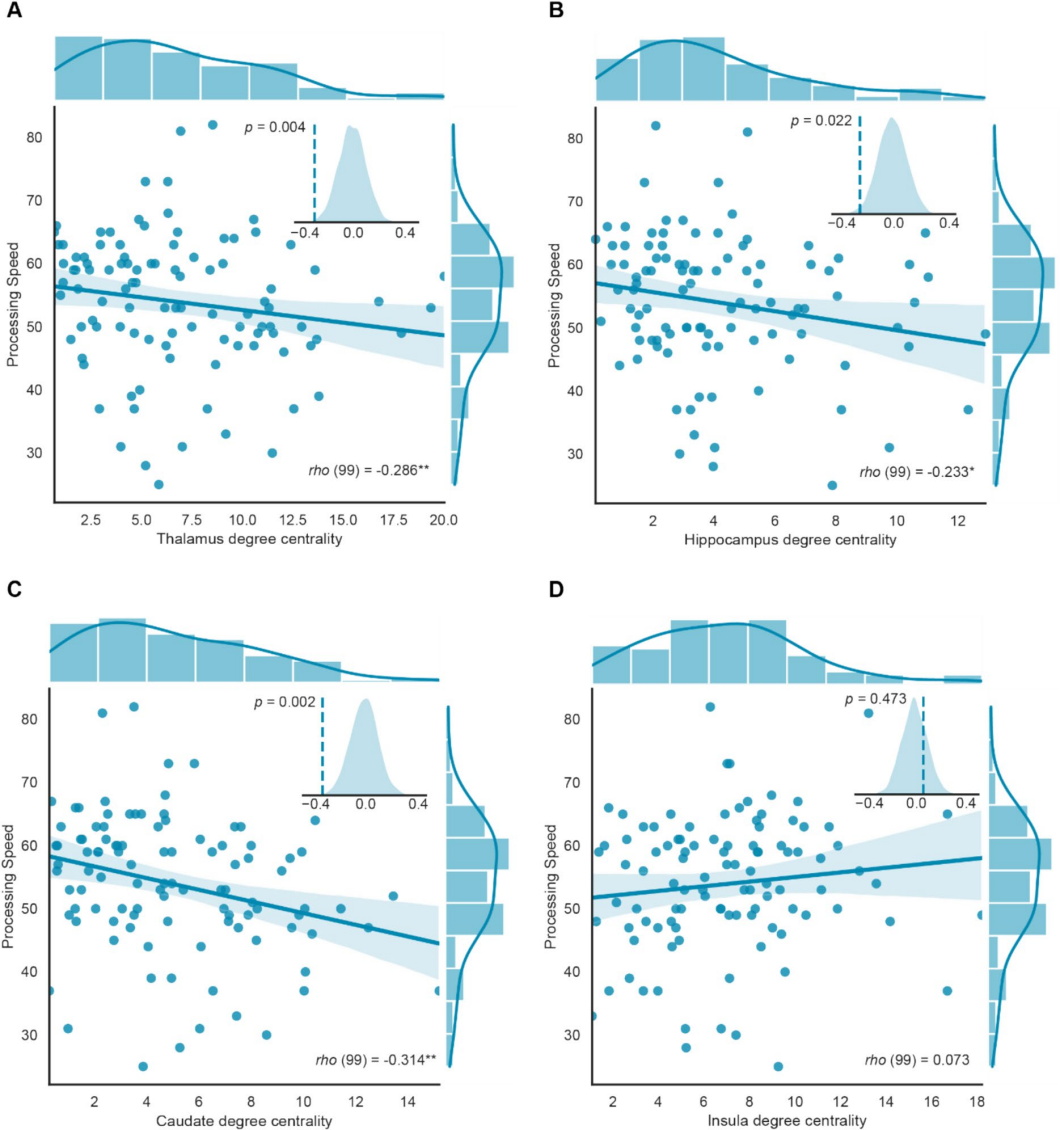
Association between intrinsic degree centrality and processing speed in older adults. Partial Spearman correlation analyses, controlling for age, sex, and years of education, were conducted to measure the relationship between intrinsic degree centrality of the ROIs and processing speed. **A** A significant negative correlation between thalamus degree centrality and processing speed in older adults (*rho* =  − 0.29, *p* = 0.004). **B** Hippocampus degree centrality had a significant negative correlation with processing speed in older adults (*rho* =  − 0.23, *p* = 0.021). **C** Caudate degree centrality had a significant negative correlation with processing speed in older adults (*rho* =  − 0.31, *p* = 0.002). **D** Insula degree centrality had no correlation with processing speed in older adults

To test whether intrinsic FC in each ROI is related to processing speed, partial correlations between degree centrality of each ROI and processing speed were conducted, controlling for age, sex, and education years. After FDR correction, we observed that the intrinsic degree centrality in thalamus (*rho* =  − 0.29, *p* = 0.004, *q* = 0.008), hippocampus (*rho* =  − 0.23, *p* = 0.021, *q* = 0.028), and caudate (*rho* =  − 0.31, *p* = 0.002, *q* = 0.008) showed significant negative correlations with processing speed but insula did not (*rho* = 0.073, *p* = 0.478, *q* = 0.478) (Table 3, Fig. 1). To further validate whether the correlation results were random, we employed a permuted partial Spearman correlation by randomly shuffling the relationship between degree centrality in thalamus, hippocampus, and caudate and processing speed 5000 times and recalculate their partial Spearman correlations. This permutation generates a distribution of randomized partial correlation coefficients, which is used to evaluate if the actual partial correlation coefficient falls within the range of random distribution. By comparing the actual partial correlation coefficient to the randomized distribution plot, we observed that the actual correlation between degree centrality in the thalamus, hippocampus, and processing speed was located at the extreme ends of the random distribution; we then assumed that the correlation was unlikely to be randomly generated (Fig. 1B). These findings confirmed that the degree centrality of the thalamus, hippocampus, and caudate was negatively correlated with processing speed. Besides, the correlations of degree centrality in other brain regions from AAL atlas are found in SI Table S1.

**Table 3:** Partial Spearman correlation results of the ROI’s degree centrality between processing speed and resilience

Partial Spearman correlation	Processing speed			Resilience		
Degree centrality	*rho*	*p*	*q* (FDR)	*rho*	*p*	*q* (FDR)
Thalamus	− 0.286	0.004	0.008	− 0.297	0.003	0.012
Hippocampus	− 0.233	0.021	0.028	− 0.089	0.383	0.51
Caudate	− 0.314	0.002	0.008	− 0.278	0.006	0.012
Insula	0.073	0.478	0.478	0.017	0.87	0.87

Note: The partial Spearman correlation controlled for age, sex, and education

### Association between intrinsic thalamus and caudate degree centrality and psychological resilience in older adults

**Fig. 2:**
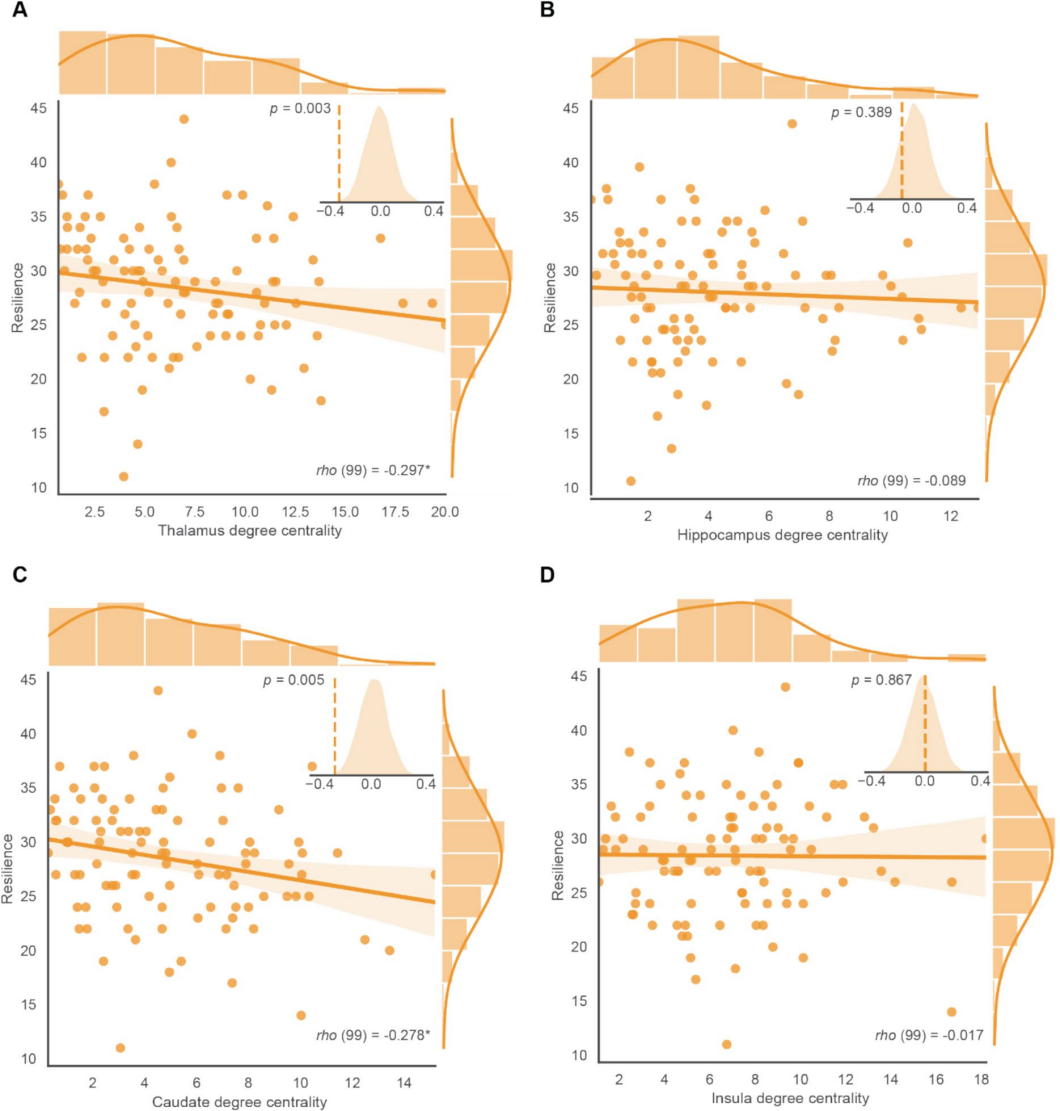
Association between psychological resilience and degree centrality of ROIs in older adults. Partial Spearman correlation analyses, controlling for age, sex, and education, were conducted to measure the relationship between intrinsic degree centrality of the ROIs and psychological resilience. **A**, **C** Significant negative correlations between thalamus (*rho* =  − 0.30, *p* = 0.003), caudate degree centrality (*rho* =  − 0.28, *p* = 0.006), and psychological resilience. **B**, **D** Hippocampus and insula degree centrality showed no significant correlation with psychological resilience

To test the association between ROIs’ intrinsic FC with resilience, partial correlations between the degree centrality of each ROI and resilience were conducted, controlling for age, sex, and education. Our analyses revealed that both thalamus (*rho* =  − 0.30, *p* = 0.003, *q* = 0.012) and caudate (*rho* =  − 0.28, *p* = 0.006, *q* = 0.012) showed significant negative correlations with resilience in older adults after FDR correction, but hippocampus (*rho* =  − 0.089, *p* = 0.383, *q* = 0.51) and insula (*rho* = 0.02, *p* = 0.87, *q* = 0.87) did not show correlation with resilience (Table 3, Fig. 2). Furthermore, another permutation analysis was also conducted for validation. The actual correlation coefficient between thalamus and caudate degree centrality and psychological resilience was observed located at the extreme ends of the random distribution, suggesting that this correlation is unlikely to be random. The correlation between nodal efficiency and betweenness centrality and resilience is found in SI Table S2, S3. This result indicates that the total FC of the thalamus and caudate represented by the index of degree centrality is associated with the level of psychological resilience in older adults. Specifically, the smaller the thalamus and caudate degree centrality, the higher the resilience in older adults.

### Intrinsic thalamus and caudate degree centrality mediated the relationship between resilience and processing speed

**Fig. 3:**
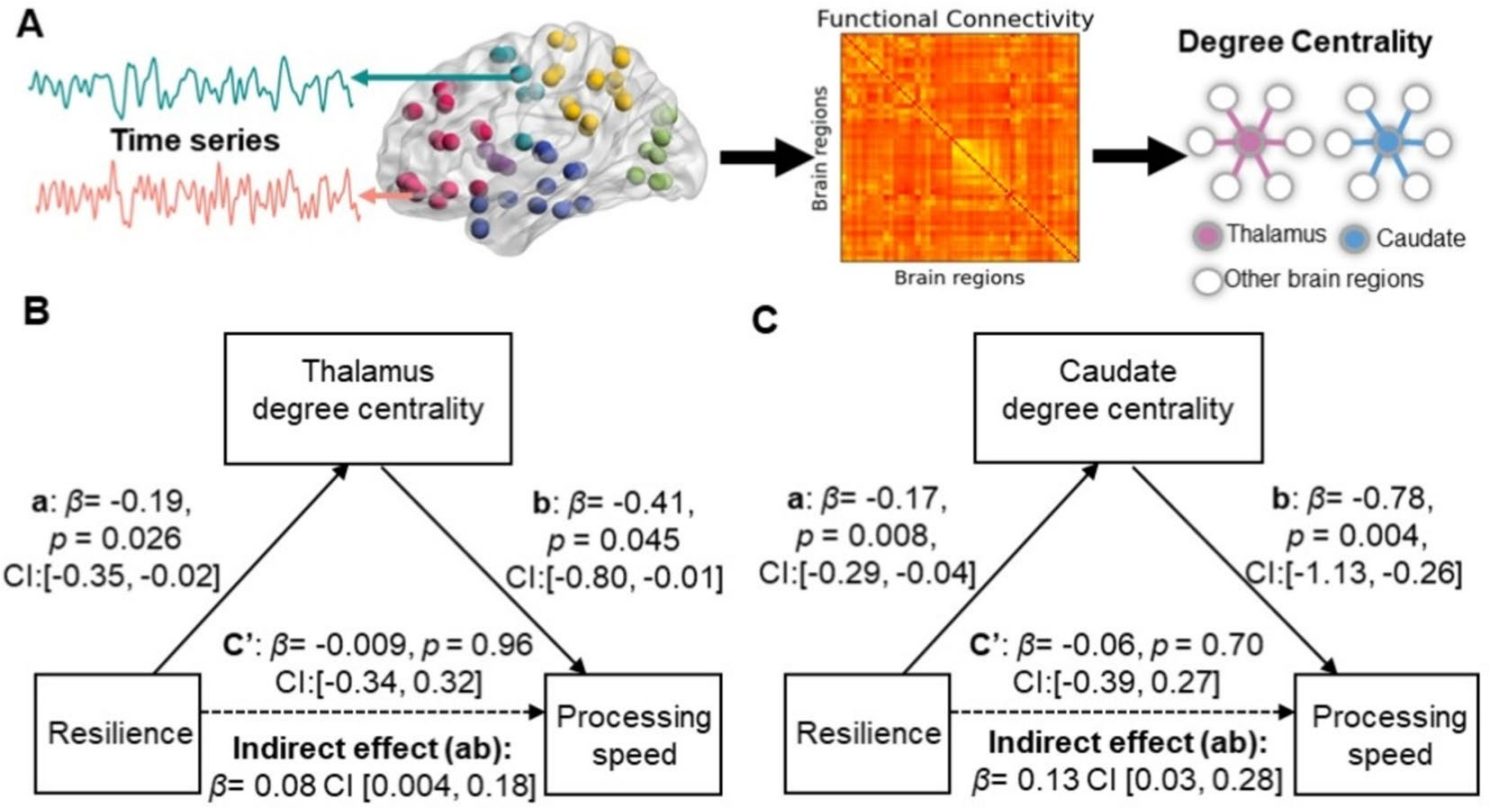
Psychological resilience mediates processing speed via the intrinsic degree centrality of the thalamus and caudate in older adults. **A** A brief flowchart of functional network calculation and the calculation of intrinsic degree centrality. **B** A mediation model shows the mediatory effect of intrinsic thalamus degree centrality that could account for an indirect association between psychological resilience and processing speed in older adults (*β* = 0.08, *p* < 0.05, 95% CI (0.004, 0.18)). **C** A mediation model shows the mediatory effect of intrinsic caudate degree centrality that could account for an indirect association between psychological resilience and processing speed in older adults (*β* = 0.13, *p* < 0.05, 95% CI (0.03, 0.28))

Additionally, we conducted a mediation analysis to understand the relationship between resilience, processing speed, and intrinsic degree centrality. Mediation analyses demonstrated that both the thalamus (indirect estimate = 0.08, 95% CI = (0.004, 0.18)) and caudate degree centrality (indirect estimate = 0.13, 95% CI = (0.03, 0.28)) significantly mediated the association between resilience and processing speed in older adults (Fig. 3B, C). The direct effects were found to be insignificant after incorporating the degree centrality of both the thalamus and caudate as mediators. The mediation effect of caudate nodal efficiency is found in SI Fig. S2. The finding suggests that the relationship between resilience and processing speed is fully explained by the degree centrality of the two brain regions.

### Exploring the potential mediating effect of education

Additionally, to further examine whether education has a potential mediating effect in the association between resilience and processing speed, we conducted a mediation model analysis. In the model, resilience was set as the independent variable (*X*), education served as the mediator (*M*), and processing speed was treated as the dependent variable (*Y*), with age and sex considered as covariates. Results revealed that education plays a significant mediating role (indirect estimate = 0.22, 95% CI = (0.07, 0.39)) in the relationship between resilience and processing speed among the ageing population. This result suggests the importance of considering educational factors when examining cognitive performance and resilience in an ageing population.

## Discussion

In this study, we investigated the relationship between psychological resilience and processing speed in older adults and the mediating roles of four ROIs identified from the literature. There was an association observed between psychological resilience and processing speed. However, this correlation was no longer significant when controlling for education, suggesting that the correlation was mainly driven by education. Nonetheless, the network analysis of the resting-state fMRI data revealed that both the thalamus and caudate degree centrality showed negative correlations with resilience and processing speed independently. A further mediation model indicated that both the thalamus and caudate degree centrality played a crucial role in influencing the relationships between resilience and processing speed in older adults. These findings highlight the importance of the intrinsic thalamus and caudate degree centrality in connecting resilience with fundamental cognitive function in an ageing population and further suggest that the segregation during information communication in subcortical regions may significantly influence cognitive performance.

### Psychological resilience shows an indirect association with processing speed

At the behavioral level, we observed that the psychological resilience of healthy older people exhibited an associative relationship with processing speed. However, when controlled for education in the partial correlation analysis, the correlation between processing speed and psychological resilience was no longer significant. Indeed, we found a significant positive association between resilience and education in our study. Prior research has posted that early life education has been proposed to directly affect psychological resilience through neuronal mechanisms and indirectly by being associated with various lifestyle factors, such as physical and social activity, as well as socioeconomic status [32, 58]. Years of education are highly correlated with the level of psychological resilience. In parallel, early life education was also found to serve as a protective factor which helps to maintain a high level of cognitive functions and delay cognitive declines in healthy older populations [71]. Our results suggest that psychological resilience in healthy older individuals may affect the performance of processing speed to a certain extent. These findings are consistent with previous reports [65, 72], documenting that higher psychological resilience is associated with better neurocognitive performance.

It is important to mention that we also observed a potential impact of resilience on processing speed through the mediation of education. Considering the strong correlation between resilience and education, additional studies are necessary to investigate potential causal links between them. Our behavioral findings uncovered the possible influence of resilience on processing speed among older adults, along with education affecting the maintenance of cognitive functions. Future research should continue to explore the relationship between psychological resilience and cognition in older adults, taking the impact of education and other potential influencing factors into account.

### Intrinsic degree centrality of thalamus and caudate negatively correlates with processing speed

Based on graph theory, degree centrality is represented by the overall functional connectivity of a certain brain node in the network, indicating the integration role of this node. In this study, we found that the intrinsic degree centrality of the thalamus, hippocampus, and caudate is all negatively correlated with processing speed in healthy older people. However, the insula degree centrality did not show a correlation with processing speed in our findings. These suggest that less intrinsic overall FC of the thalamus, hippocampus, as well as caudate, represented by their degree centrality, is associated with better processing speed. The global number of functional connections from the thalamus was found to be significantly associated with episodic memory function in a sample of healthy older adults [21]. Research focused on processing speed in later-life depression found that the decreased FC between the hippocampus and middle frontal gyrus is involved in the processing speed [40]. And the caudate has been previously identified as a vital role in engaging in the active neural circuit associated with various cognitive functions, including switching attention between processes, processing speed, and mental flexibility [28, 70]. Therefore, our findings are consistent with prior research and also provide further evidence of the involvement of the thalamus and caudate during processing speed in the healthy older population. One possible explanation for the negative correlation could be that reduced intrinsic FC of the thalamus and caudate allows for more efficient allocation of cognitive resources required for processing speed [34]. Besides, both the thalamus and caudate serve as critical components in the cortico-striatal-thalamo-cortical network, which subserve at different cognitive hierarchies [37]. The observed reduction in intrinsic degree centrality in these subcortical regions further suggests that information processing speed is a fundamental aspect of cognitive function [18, 80].

It is also worth considering that the caudate connects structurally and functionally with areas of the prefrontal cortex such as the dorsal and medial prefrontal cortex [12, 55, 62]. Based on previous theory [51, 57], we thus further speculate that two possible mechanisms might be relevant to account for our observed correlation between the intrinsic reduction in caudate degree centrality and better processing speed. One possible mechanism is functional specialization, where the decreased functional connectivity of the caudate may be a neural signature of more efficient cognitive function. Existing research has found that older individuals with more distinctive large-scale networks performed more efficiently during working memory tasks [36]. The functional dissociation between different networks might be beneficial for transferring information and maintaining better performance in cognition. Alternatively, the negative correlations between caudate degree centrality and resilience and processing speed may reflect the brain’s flexibility to compensate for age-related cognitive decline [7]. Specifically, older individuals with lower resilience may need to recruit additional neural resources and more subcortical regions’ (thalamus and caudate) engagement to maintain optimal cognitive performance when facing the challenges of ageing. However, further research is needed to explore the specific neural pathways and mechanisms that underlie the relationship between caudate functional connectivity, processing speed, and resilience. Such investigations could provide valuable insights into the role of the caudate and its FC in cognitive ageing and help advance our understanding of the neural basis of resilience.

### Psychological resilience affects processing speed via intrinsic thalamus and caudate degree centrality

In conjunction with the association between processing speed and intrinsic degree centrality in both the thalamus and caudate, we further observed that psychological resilience also showed a negative correlation with intrinsic degree centrality in the thalamus and caudate. However, we did not observe an association between degree centrality in the hippocampus and insula with resilience in this study. These findings demonstrated that the individuals with higher resilience exhibited a lower degree centrality in both the thalamus and caudate intrinsically, reflecting that psychological resilience is associated with the segregation of these two subcortical regions from the rest of the brain networks [12, 57]. The thalamus projects to the cortex, including frontal, superior, and dorsal cortical regions in the widely acknowledged parallel loop model of subcortical connections [21]. As one of the key components in stress-related responses, the thalamus was found to be more active in response to stress [33, 69]. Previous research revealed that changes in thalamus activation as well as increased thalamocortical integration were observed after stressor exposure [30]. Lower intrinsic thalamus degree centrality means fewer or weaker functional connections with the rest brain regions. The explanation for the less connected thalamus is linked with higher resilience in older adults, may be that individuals with high resilience are under low levels of stress and the thalamus is more segregated from the rest brain regions [29]. Previous research has identified the caudate as the most age-sensitive region rich in dopamine receptors [38] and the dopamine receptors served as strong mediators of age-related cognitive decline [36]. The observed correlation between increased resilience and decreased caudate degree centrality could be due to changes in the caudate’s intrinsic functional connections in individuals who are more resilient. This adjustment might indicate a decreased reliance or need for compensation from the dopamine system to maintain cognitive function while dealing with the challenge of ageing [55]. Consequently, individuals with greater psychological resilience are less likely to experience the increase in dopamine-related cortical-caudate connections related to dopamine that typically occurs with ageing.

Critically, we observed that both the intrinsic thalamus and caudate degree centrality mediated the indirect association between psychological resilience and processing speed in older adults. These complement our results suggesting that a higher level of psychological resilience leads to better processing speed via lower intrinsic thalamus and caudate degree centrality in healthy older adults. Together with prior findings, our study further elucidates the relationship between psychological resilience, intrinsic FC of the thalamus and caudate, and processing speed in healthy ageing individuals, emphasizing the significant protective role of psychological resilience in maintaining brain function and cognition of the older population to reduce ageing-related cognitive decline. It should be noted that our sample has a male to female ratio imbalance. When we conducted separate analyses for each subgroup, the mediation models remained significant only in the female subgroup and not in the male subgroup. This suggests that while our findings hold strong for older female adults, caution should be exercised when applying these results to the entire population. Future studies should aim to recruit a larger number of male participants to further investigate these findings. These findings also offer potential targets of observing the functional activation and connectivity in the thalamus and caudate and enhance the level of psychological resilience for interventions aimed at enhancing cognitive function in older adults.

## Limitations

There are several limitations to be considered in the current study. First, a potential limitation of our study is the male to female ratio imbalance in our sample, with a larger proportion of females than males. Second, our study solely investigated the mediating effect of intrinsic FC on resilience and processing speed, without considering other potential mediation models. Alternative possibilities could exist, such as processing speed mediating resilience through brain FC. More research is encouraged to explore various potential combinations for constructing mediation models to gain a comprehensive understanding of the relationship among processing speed, intrinsic FC, and resilience. Third, while our study did identify significant mediation effects, the effect sizes were relatively small. To better establish the mediation effects, future research should consider recruiting a larger sample size. Lastly, the cross-sectional design also limited us from establishing causal relationships between psychological resilience, the brain FC, and processing speed in the elderly. Future longitudinal design studies are needed to investigate the association between psychological resilience and cognitive function and uncover the causal relationship between psychological resilience and cognitive functions in ageing people.

## Conclusions

In conclusion, our study reveals that there is no direct association between psychological resilience and processing speed in healthy older adults. However, we did find a negative association between psychological resilience and intrinsic thalamus and caudate FC. Moreover, it seems that psychological resilience may contribute to the enhancement of processing speed via the decrease of intrinsic thalamus and caudate degree centrality. These findings provide valuable insights into the role of psychological resilience in protecting the cognitive functions among the ageing population, and the underpinning brain mechanisms of the resilience effect on processing speed, which could inform future research to explore potential interventions aimed at boosting psychological resilience as a means to mitigate age-related cognitive decline.

## Supplementary Information

Below is the link to the electronic supplementary material.

Supplementary file1 (DOCX 181 KB)

## Data Availability

The anonymized minimal dataset supporting the conclusions of this study can be obtained upon reasonable request from the corresponding authors, as participants did not provide consent for public sharing of the raw data.
