# Supplementary material for: Intrinsic brain functional connectivity mediates the relationship between psychological resilience and cognitive decline in ageing
Source: GeroScience. 2025 Feb 3;47(4):5635–50. doi: 10.1007/s11357-025-01529-5 (PMC12397464; doi:10.1007/s11357-025-01529-5)
Supplement: Supplementary file 1 — Supplementary file1 (DOCX 181 KB) [file 11357_2025_1529_MOESM1_ESM.docx]

**Supplementary Information for**

**Intrinsic brain functional connectivity mediates the relationship between psychological resilience and cognitive decline in ageing**

Menglu Chen ^1,2^, Mengxia Gao^1,2^, Junji Ma^1,2^, Tatia M.C. Lee ^1,2*^

^1^ State Key Laboratory of Brain and Cognitive Sciences, The University of Hong Kong, Hong Kong SAR, China

^2^ Laboratory of Neuropsychology & Human Neuroscience, The University of Hong Kong, Hong Kong SAR, China

***Correspondence to:**

Tatia M.C. Lee, Ph.D.

Address: Room 656, Laboratory of Neuropsychology & Human Neuroscience, The Jockey Club Tower, The University of Hong Kong, Pokfulam, Hong Kong SAR, China

Tel.: 852-39178394

E-mail address: tmclee@hku.hk

**Exploring the potential brain regions reaming in the AAL related to processing speed**

To further investigate whether additional brain regions might be related to processing speed, we conducted a partial Spearman correlation to examine the relationship between processing speed and the degree centrality of all regions from the AAL atlas, with age, sex and education as covariates.

The results of these analyses demonstrated that, besides the degree centrality of the four ROIs, the degree centrality of the right Gyrus rectus (*rho* = 0.23, *p* = 0.026), right Inferior occipital gyrus (*rho* = 0.22, *p* = 0.034) and right Putamen (*rho* = 0.21, *p* = 0.036) showed significant correlations with processing speed when age, sex and education were included as covariates (see Table S1).

Furthermore, to explore whether the degree centrality of these brain regions is also related to resilience, we conducted another spearman correlation analysis controlling for age, sex and education. However, we did not observe any correlation between these three brain regions with resilience (see Table S1).

**Table S1**. Partial spearman correlation results of brain regions between resilience and degree centrality

| Partial spearman correlation | Processing speed | | | Resilience | | |
| --- | --- | --- | --- | --- | --- | --- |
| Degree centrality | *rho* | *p* | covariates | *rho* | *p* | covariates |
| right Gyrus rectus | 0.23 | 0.026 | Age, sex and education | - 0.186 | 0.066 | Age, sex and education |
| right Inferior occipital gyrus | 0.22 | 0.034 |  | 0.091 | 0.374 |  |
| right Putamen | -0.21 | 0.036 |  | -0.13 | 0.202 |  |

**
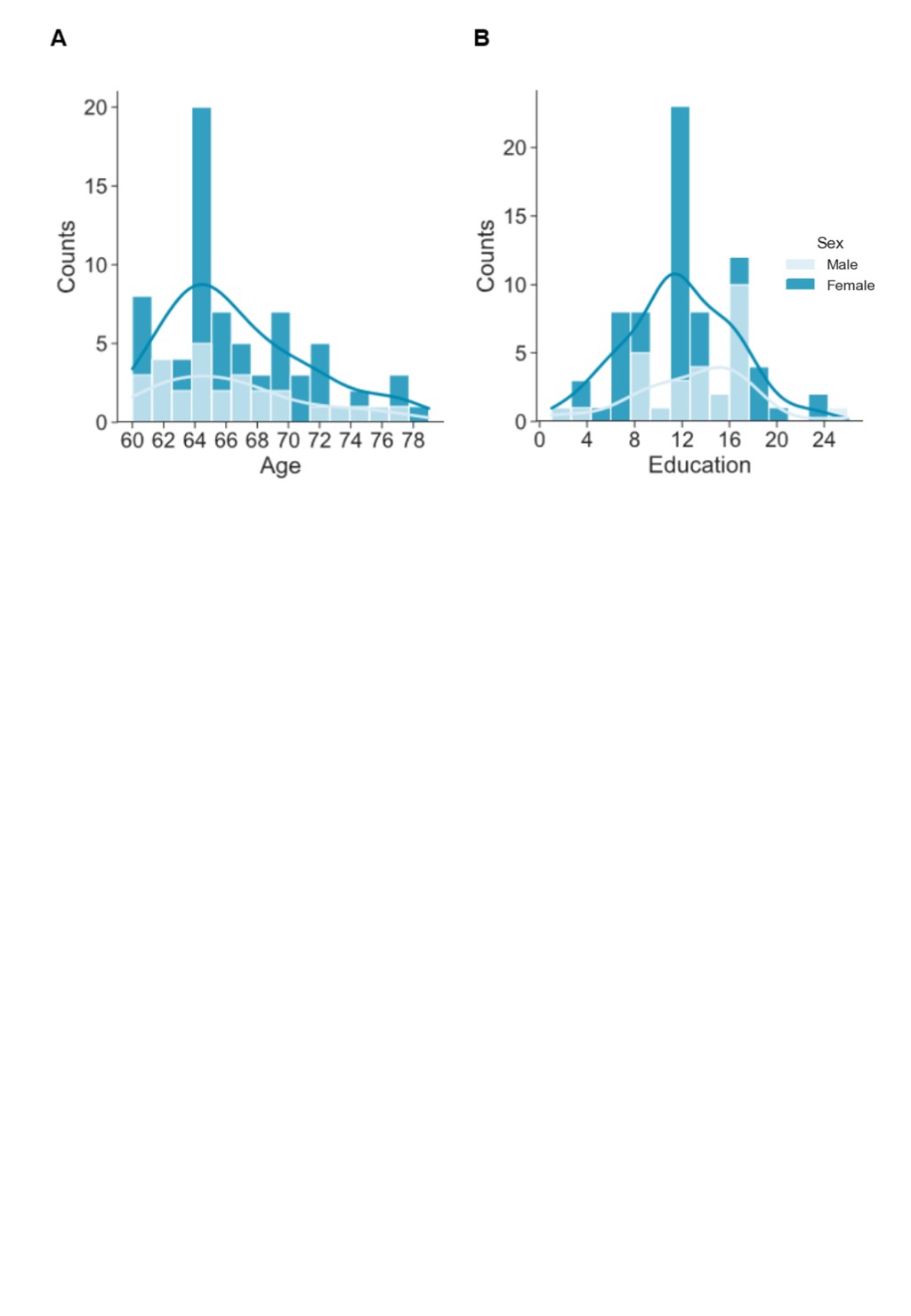
**

**Figure.S1. Demographics of participants**: (A) A total of 102 older adults, ranging in age from 60 to 79, met the inclusion criteria for the final analysis. (B) The participants' years of education varied between 1 and 26 years.

**Exploring more measurements based on graph theory.**

To explore whether other measures from graph theory of the four regions of interest would be related to processing speed and resilience, we conducted more nodal based graph theory measurements including nodal efficiency and betweenness centrality. Nodal efficiency reflects the efficiency of parallel information transfer of this specific brain region to all other nodes in the network. Nodal efficiency is calculated as the mean of the inverse shortest path length from the specific brain region to all other nodes, which represents how efficiently a brain region communicates with the rest of the brain (Uehara et al., 2014; Wang et al., 2015). Betweenness centrality reflects the influence of a specific brain region on information flow to all other nodes, defined as the fraction of all shortest paths in the network that pass through that node (Zhuang et al., 2022). Unlike degree centrality, which simply counts direct connections, these two addition measurements emphasize the dynamics of information transfer of specific brain regions within the neural network.

Similar to the calculation of degree centrality, the nodal efficiency and betweenness centrality of the four regions of interest (ROIs) were also computed using the GRETNA toolbox (https://www.nitrc.org/projects/gretna/). A weighted network approach was employed and included only the positive correlation values between nodes. To maintain consistency with the degree centrality calculations, we applied a range of sparsity thresholds from 0.05 to 0.5 in increments of 0.05, averaging the results across these 10 thresholds to ensure a robust and comprehensive assessment of network properties (Power et al., 2011; Xiao et al., 2019). Additionally, we combined the nodal efficiency and betweenness centrality values extracted from both the left and right hemispheres by averaging their respective values.

To further investigate the relationship between nodal efficiency and betweenness centrality in each ROI with respect to processing speed and resilience. We conducted partial correlations while controlling for age, sex, and education. Correlation results after false discovery rate (FDR) correction demonstrated that the nodal efficiency in caudate showed significant correlations with both processing speed (*rho* = -0.275, *p* = 0.006, *q* = 0.024), and resilience (*rho* = -0.268, *p* = 0.008, *q* = 0.016), with age, sex and education included as covariates (see Table S2).

**Table S2**. Partial Spearman correlation results of the ROI’s nodal efficiency between processing speed and resilience.

| Partial spearman correlation | Processing speed | | | Resilience | | |
| --- | --- | --- | --- | --- | --- | --- |
| Nodal efficiency | *rho* | *p* | *q* (FDR) | *rho* | *p* | *q* (FDR) |
| Thalamus  Hippocampus | - 0.213 | 0.034 | 0.068 | - 0.283 | 0.005 | 0.02 |
|  | -0.163 | 0.107 | 0.143 | - 0.107 | 0.293 | 0.39 |
| Caudate | - 0.275 | 0.006 | 0.024 | - 0.268 | 0.008 | 0.016 |
| Insula | 0.08 | 0.43 | 0.43 | -0.031 | 0.76 | 0.76 |

Note: The partial spearman correlation controlled for age, sex and education.

However, we did not observe any correlation between each ROIs’ betweenness centrality and processing speed, resilience (see Table S3).

**Table S3**. Partial spearman correlation results of ROIs’ betweenness centrality between processing speed and resilience.

| Partial spearman correlation | Processing speed | | | Resilience | | |
| --- | --- | --- | --- | --- | --- | --- |
| Betweenness centrality | *rho* | *p* | *q* (FDR) | *rho* | *p* | *q* (FDR) |
| Thalamus  Hippocampus | - 0.131 | 0.196 | 0.392 | - 0.156 | 0.126 | 0.252 |
|  | 0.046 | 0.654 | 0.654 | - 0.04 | 0.695 | 0.695 |
| Caudate | - 0.097 | 0.34 | 0.453 | - 0.115 | 0.259 | 0.345 |
| Insula | 0.171 | 0.09 | 0.36 | 0.194 | 0.055 | 0.22 |

Based on the observation above, we further conducted a mediation analysis to explore whether nodal efficiency in caudate mediates the relationship between resilience processing speed. In the mediation model, age, sex and education were included as covariates. The results indicated that the nodal efficiency in caudate significantly mediated the relationship between resilience and processing speed in older adults (*β* = 0.1, *p* <0.05, 95%CI [0.01, 0.24]) (see Fig.S2).

**
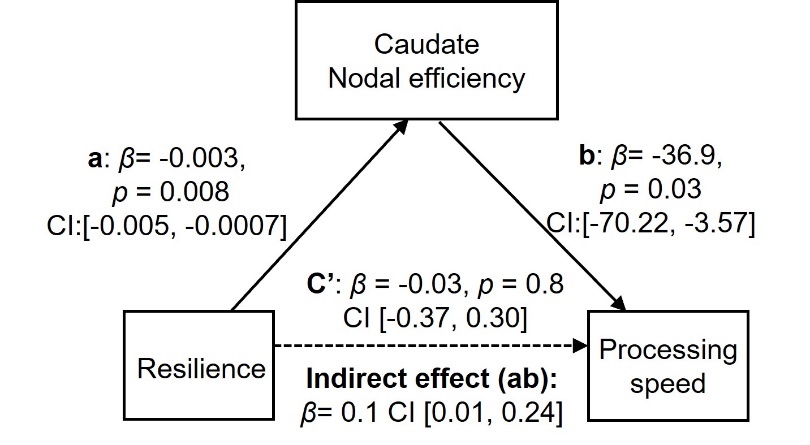
**

**Figure.S2. Resilience mediated processing speed via caudate nodal efficiency in older adults.** A mediation model showed the mediatory effect of caudate nodal efficiency that could account for an indirect association between resilience and processing speed in older adults (*β* = 0.1, 95%CI [0.01, 0.24]).

The findings on nodal efficiency in caudate were consistent with our results regarding caudate degree centrality. While degree centrality reflects the total functional connectivity between a specific brain node and the other brain nodes, higher degree centrality in caudate indicated that it was connected with many regions in the whole brain. Nodal efficiency, on the other hand, measures how efficiently information can be transferred from this specific brain regions to other nodes. Higher nodal efficiency in caudate suggested that it can quickly and effectively share information with other brain regions. Together, these two approaches provided different perspectives on caudate’s role based on graph theory, further validating the crucial involvement of caudate in processing speed and resilience.

**Exploring the potential effects of sex difference**

To control the potential confounding effect of male to female ratio imbalance, we conducted two sample t-tests to examine whether the female and male subgroups had significant differences in the variables of interest including resilience, processing speed and the degree centrality in the 4 ROIs. Results demonstrated that there were no sex differences among these variables (see Table S4 below).

To further confirm our results, we repeated all our analyses separately in the two sex subgroups (Female:73; Male: 28). First, we conducted the subgroup Spearman correlation analyses and the results demonstrated that in the female subgroup thalamus and caudate are still correlated with both processing speed (thalamus: rho = -0.30, p = 0.01, caudate: rho = -0.34, p = 0.004), and resilience (thalamus: rho = -0.27, p = 0.02, caudate: rho = -0.24, p = 0.04), while the correlation between processing speed and hippocampus (rho = -0.20, p = 0.09) was no significant. In male subgroup, thalamus and caudate were significantly correlated with resilience (thalamus: rho = -0.44, p = 0.02, caudate: rho = -0.39, p = 0.04), but not with processing speed (thalamus: rho = -0.20, p = 0.34, caudate: rho = -0.18, p = 0.38, hippocampus: rho = -0.32, p = 0.11). Furthermore, we conducted mediation model analysis for each sex subgroups while controlling for age and education. Results demonstrated that only the caudate (indirect estimate = 0.13, 95% CI = [0.004, 0.33]) but not the thalamus (indirect estimate = -0.08, 95% CI = [-0.002, 0.22]) significantly mediated the relationship between resilience and processing speed in the female subgroup. In the male subgroup, no significant mediation effect was observed.

**Table S4:** The t test results of sex differences between two sex sample among the variables of resilience, processing speed and the degree centrality in four ROIs.

| T test between sex subgroups | *t* | *df* | *P* _value | Effect size |
| --- | --- | --- | --- | --- |
| Resilience | -0.543 | 99 | 0.59 | -0.12 |
| Processing speed | 0.71 | 99 | 0.48 | 0.16 |
| Thalamus degree centrality | -0.88 | 99 | 0.38 | -0.20 |
| Hippocampus degree centrality | -0.95 | 99 | 0.35 | -0.21 |
| Caudate degree centrality | 0.38 | 99 | 0.71 | 0.08 |
| Insula degree centrality | -1.5 | 99 | 0.14 | -0.33 |

**References**

Power, J. D., Cohen, A. L., Nelson, S. M., Wig, G. S., Barnes, K. A., Church, J. A., Vogel, A. C., Laumann, T. O., Miezin, F. M., Schlaggar, B. L., & Petersen, S. E. (2011). Functional Network Organization of the Human Brain. *Neuron*, *72*(4), 665–678. https://doi.org/10.1016/j.neuron.2011.09.006

Uehara, T., Yamasaki, T., Okamoto, T., Koike, T., Kan, S., Miyauchi, S., Kira, J. -i., & Tobimatsu, S. (2014). Efficiency of a “Small-World” Brain Network Depends on Consciousness Level: A Resting-State fMRI Study. *Cerebral Cortex*, *24*(6), 1529–1539. https://doi.org/10.1093/cercor/bht004

Wang, J., Wang, X., Xia, M., Liao, X., Evans, A., & He, Y. (2015). GRETNA: A graph theoretical network analysis toolbox for imaging connectomics. *Frontiers in Human Neuroscience*, *9*, 386. https://doi.org/10.3389/fnhum.2015.00386

Xiao, F., Lu, C., Zhao, D., Zou, Q., Xu, L., Li, J., Zhang, J., & Han, F. (2019). Independent Component Analysis and Graph Theoretical Analysis in Patients with Narcolepsy. *Neuroscience Bulletin*, *35*(4), 743–755. https://doi.org/10.1007/s12264-018-0307-6

Zhuang, L., Wang, J., Xiong, B., Bian, C., Hao, L., Bayley, P., & Qin, S. (2022). Rapid neural reorganization during retrieval practice predicts subsequent long-term retention and false memory. *Nature Human Behaviour*, *6*, 1–12. https://doi.org/10.1038/s41562-021-01188-4
